# Protective effect of Tribulus *terrestris* fruit extract on cerulein-induced acute pancreatitis in mice

**Published:** 2017

**Authors:** Mina Borran, Mohsen Minaiyan, Behzad Zolfaghari, Parvin Mahzouni

**Affiliations:** 1 *School of Pharmacy and Pharmaceutical Sciences, Isfahan University of Medical Sciences, Isfahan, Iran*; 2 *Department of Pharmacology and Isfahan Pharmaceutical Sciences Research Center, School of Pharmacy and Pharmaceutical Sciences, Isfahan University of Medical Sciences, Isfahan, Iran*; 3 *Department of Pharmacognosy, School of Pharmacy and Pharmaceutical Sciences, Isfahan University of Medical Sciences, Isfahan, Iran *; 4 *Department of Clinical Pathology, School of Medicine, Isfahan University of Medical Sciences, Isfahan, Iran*

**Keywords:** Acute pancreatitis, Inflammation, T.terrestris, Cerulein, Mice

## Abstract

**Objective:**

Antioxidant, anti-inflammatory, analgesic and antimicrobial activities of *Tribulus terrestris* (*T. terrestris*) could be helpful in the treatment of acute pancreatitis; thus, this study was designed to investigate the effects of *T. terrestris *on cerulein-induced acute pancreatitis in mice.

**Materials and Methods:**

Three doses (100, 200 and 400 mg/kg) of *T. terrestris *hydro-alcoholic extract were administered both orally (60 minutes before pancreatitis induction, p.o.) and intra-peritoneally (30 minutes before pancreatitis induction, i.p.) to different groups of mice (n=6). Pancreatitis was induced by ﬁve injections (i.p.) of cerulein 50μg/kg body weight with 1 hr intervals. Animals were euthanized 5 hr after the last injection of cerulein and tissue injures were assessed biochemically and pathologically.

**Results:**

*T. terrestris *extract 200 and 400mg/kg (p.o.) and *T. terrestris *extract 400 mg/kg (i.p.) reduced pancreatic tissue myeloperoxidase (MPO) activity and serum amylase and lipase levels and alleviated histological parameters.

**Conclusion:**

These data suggest that *T. terrestris *hydro-alcoholic extract was effective in protecting against experimental acute pancreatitis and possibly the efficacy depends on dose and route of administration.

## Introduction

Acute pancreatitis (AP) denotes a potentially lethal disorder which is characterized by persistent inflammation of the pancreas over a short period of time as well as elevation in serum level of digestive enzymes without specific therapy (Spanier et al., 2008 ▶).

It seems that acute pancreatitis could be potentially life threatening. The morbidity and mortality associated with pancreatitis are secondary to cardiogenic shock, cardiac, renal and respiratory insufficiency and hepatic dysfunction. Also, it can lead to progression of failure in distant organs which denotes multiple organ failure (Lankisch and Banks, 2013 ▶).

Drinking alcohol beverages, gallstones, hypertriglyceridemia, abdominal trauma, hypercalcemia, hyperparathyroidism, narrowing of pancreas duct and systemic infections are among well-established causes of acute pancreatitis (Sakorafas and Tsiotou, 2000 ▶; Wang et al., 2009 ▶).

Some drugs like ACE-inhibitors, statins, estrogens, diuretics, highly active antiretroviral treatment (HAART), and valproic acid have been also implicated in drug-induced acute pancreatitis with limited understanding of their mechanisms (Badalov et al., 2007 ▶; Kaurich, 2008 ▶).

The early pathophysiology of the acute pancreatitis has not been well understood, but some clinical studies have shown that after an initial acinar cell injury, pro-inflammatory cytokines such as interleukin (IL) 1, IL-6, IL-8 and tumor necrosis factor alpha (TNF- α) are increased in the serum of patients with acute pancreatitis (Pooran et al., 2003 ▶) while the degree of cytokine elevation correlates with disease severity (Raraty et al., 2004 ▶).

Current treatment of pancreatitis is largely supportive and consists of different combination therapy including: antibiotics, fluid resuscitation, nutritional support and pain control. Since there is no single drug or therapy known to treat this disease effectively, a great need exists to find new therapies for acute pancreatitis (Kambhampati et al., 2014 ▶).

It seems that medicinal herbs are suitable alternatives for treatment of acute pancreatitis as they have various components that can cover different aspect of pancreatitis pathophysiology as it has been shown in various studies (Abed et al., 2012 ▶; Genovese et al., 2006 ▶; Minaiyan et al., 2012 ▶; Minaiyan et al., 2014b ▶; Qiong et al., 2005 ▶).

*Tribulus terrestris *(Caltorps) is belonging to Zygophillaceae family which consists of 20 species around the world. Caltorps is an annual plant adapted to grow in dry climate locations through India, China, parts of Europe and Iran (Kianbakht and Jahaniani, 2003 ▶).

*T. terrestris *extract is well patronized in Iranian and Indian traditional medicine texts like Ayurvadic text for different medicinal uses like anti-inflammatory, nutritive, diuretic, anti-dysuria, hepatoprotective and pain alleviating purposes. The extracts of the fruits and leaves also have cardiac stimulant and fertility potentiating activity and may improve sexual activity (Chhatre et al., 2014 ▶; Neychev and Mitev, 2005 ▶). Furthermore, antioxidant, analgesic, anti-inflammatory and antibacterial properties of *T. terrestris* fractions have been demonstrated in several experimental investigations (Baburao et al., 2009; Heidari et al., 2007a ▶; Mitra et al., 2012; Zheleva-Dimitrova et al., 2012 ▶).

*T. terrestris *exhibits a promising safety profile as the maximum tolerated dose of 50% ethanol extract of fruits in mice was 100 g/kg (Shaheen et al., 2012 ▶). Its safety in humans has also been shown; so, the *T. terrestris *extract as a drug supplement is found in market (Rogerson et al., 2007 ▶).

These data suggest that *T. terrestris* might possess protective effects against pancreatitis; so, the present study was performed to evaluate the protective effects of *T. terrestris *against cerulein -induced acute pancreatitis in mice to have a better insight into the mechanism (s) of actions of *T. terrestris *on pancreatitis.

## Materials and Methods

**Plant material and extraction**

*T. terrestris *fruits were collected from the local areas of Fereydan, Isfahan province, Iran. The plant identity was confirmed by Pharmacognosy Department of School of Pharmacy, Isfahan University of Medical Sciences, Isfahan, Iran. The fruits were cleaned, dried and grinded and the dried powder was extracted with ethanol: water (70:30) using maceration method. The soaked powder was kept at room temperature for 24 hr with shaking, then, it was filtered. This process was repeated three times. Subsequently, all the filtrate was pooled, evaporated to dryness in a rotary evaporator to yield a semi-solid extract. Then, this extract was freeze-dried under reduced pressure and stored in the refrigerator till use (Hajhashemi et al., 2010 ▶).

**Determination of total phenols**

The total phenolic content of freeze-dried powders of *T. terrestris *fruit was checked by Folin-Ciocalteau micro-method by plotting the standard curve using gallic acid solutions (50, 100, 150, 250 and 500 mg/L) as reference agent.

 A total volume of 20 μl of blank, standard and sample solutions were separately added into tubes and to each tube, 1.58 mL of distilled water was added. Then, 100 μL of Folin-Ciocalteu Reagent (Sigma, St. Louis, MO, USA) was added and mixed well. After 8 min, 300 μL of sodium carbonate solution 20% was added and mixed. The solutions were maintained at 40°C for 30 min and the absorbance of solutions was detected at 760 nm against the blank using ultra violet (UV)-Vis spectrophotometer (Waterman and Mole, 1994 ▶).

**Animals**

Sixty male mice (12 weeks old, 30-40 g) were used. Mice were housed in plastic mouse cages (6 in each) with controlled temperature, humidity, and light/dark cycles (12hr/12hr) and had free access to pelleted rodent chow and tap water.

 Before initiation of the experiment, the animals were fasted over the night for 12 hr. The study was authenticated by the Ethics survey for Animal Care and Uses, Isfahan University of Medical Sciences, Isfahan, Iran.

**Induction of pancreatitis**

Acute pancreatitis was induced by ﬁve intraperitoneal (i.p.) injections of cerulein 50μg/kg body weight (Sigma, St. Louis, MO, USA) with 1 hr intervals (Michalski et al., 2007 ▶).

**Grouping**

Animals were randomly divided into the following 10 groups (n=6).

Sham group: Twelve mice were treated p.o. and/or i.p. with 10ml/kg body weight (B.W.) normal saline without any pancreatitis induction.

Control groups: Twelve mice were treated p.o. and/or i.p. with 10ml/kg B.W. normal saline 60 and 30 min before pancreatitis induction, respectively.

Oral extract treated groups: Eighteen mice were treated with 100, 200, 400 mg/kg B.W. (p.o.) of hydro-alcoholic extract of *T. terrestris *60 min before pancreatitis inductions.

Parenteral extracts treated groups: Eighteen mice were treated with 100, 200 and 400 mg/kg B.W. (i.p.) of hydro-alcoholic extract of *T. terrestris *30 min before pancreatitis induction. Then, 5 hr after the last injection of cerulein, blood samples were taken by intra-neck puncture after head blowing and stored at -20 C for biochemical analysis till 2 weeks. 

The pancreas was quickly removed and ﬁxed in formaldehyde (10%) for histological examination. Besides, portions of this organ were immediately frozen in liquid nitrogen and stored at -70^◦^C until assayed for myeloperoxidase (MPO) activity. Microscopic image was captured by a pathologist unaware about the study protocol using a professional camera (Sony®, Japan) set on an optical microscope (Minaiyan et al., 2014b ▶).

**Amylase and lipase activity analysis**

 Serum lipase and amylase activity were calculated using commercially available lipase and amylase kits (Pars-Azmoon Company, Tehran, Iran) (Minaiyan et al., 2014b ▶).

**Myeloperoxidase activity assay**

MPO activity, an index of poly-morphonuclear leukocyte accumulation and oxidative stress, was determined according to the modiﬁed method reported by Bradley et al. (1982) ▶**.**

Pancreas tissue was homogenized in 1mL of 50 mM potassium phosphate buﬀer containing 0.5% HTAB (hexadecyl trimethyl ammonium bromide). Then, the homogenate was homogenized in an ice bath for 10 s, freeze-thawed thrice with sonication between cycles.

The suspensions were centrifuged at 15,000 rpm for 15 min at 4^◦^C and then, the supernatant (0.1mL) was allowed to react with 2.9 mL of 50 mM potassium phosphate buﬀer (pH 6.0) containing O-dianisidine dihydrochloride (0.167mg/mL) and 0.005% hydrogen peroxide. 

The absorbance of the reaction mixture was measured at 450nm using a UV-Vis spectrophotometer. MPO activity was expressed in units (U) per gram of wet tissue weight (Minaiyan et al., 2014a ▶).

**Histological Examination**

Paraﬃn-embedded pancreas samples were sectioned (5μm), and stained with hematoxylin and eosin (H and E). The histological grading of edema was done using a scale ranging from 0 to 3 (0=no edema, 1=interlobular edema, 2=interlobular and moderate intralobular edema and 3=interlobular edema and severe intralobular edema). 

Leukocyte inﬁltration was also graded from 0 to 3 (0=absent, 1=scarce perivascular inﬁltration, 2=moderate perivascular and scarce diﬀuse inﬁltration, and 3=abundant diﬀuse inﬁltration). 

Grading of vacuolization was based on the appropriate percentage of acinar cells involved 0=absent, 1=less than 25%, 2=25–50%, and 3=more than 50% of acinar cells (Dembiński et al., 2008 ▶).

**Statistical Analysis**

In this study, all the statistical analyses were performed by GraphPad Prism ver 5.04. Biochemical quantitative data are expressed as mean±SEM. Graded data are expressed as median (range) values. Statistical analysis was carried out using one-way analysis of variance (ANOVA) followed by Tukey’s multiple comparison tests. Nonparametric data was analyzed by Mann-Whitney U test and p<0.05 was considered as significant.

## Results

**Total phenolic content**

Total phenol content based on Gallic acid equivalency (GAE) was 52.9 ± 2.3 mg GAE/g.

**Effects of** ***T. terrestris*** **extract on the serum levels of amylase and lipase**

**Figure 1:**
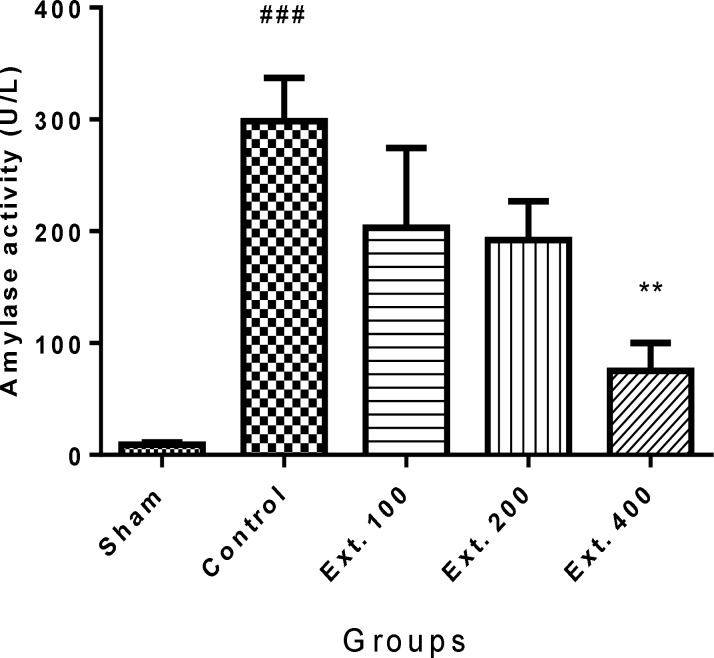
Effect of T. terrestris fruits hydro-alcoholic extract on serum amylase activity (U/L) of cerulien-induced acute pancreatitis in mice (i.p.). Sham: normal mice treated with normal saline (10 mL/kg); Control: received cerulein (50 µg/kg) and treated with normal saline (10 mL/kg), Ext. 100, 200, 400: received cerulein (50 µg/kg) and treated with extract, i.p.. Data are shown as mean±SEM of six animals in each group. ^**^p<0.01 versus control, ^###^p<0.001 versus sham

**Figure 2:**
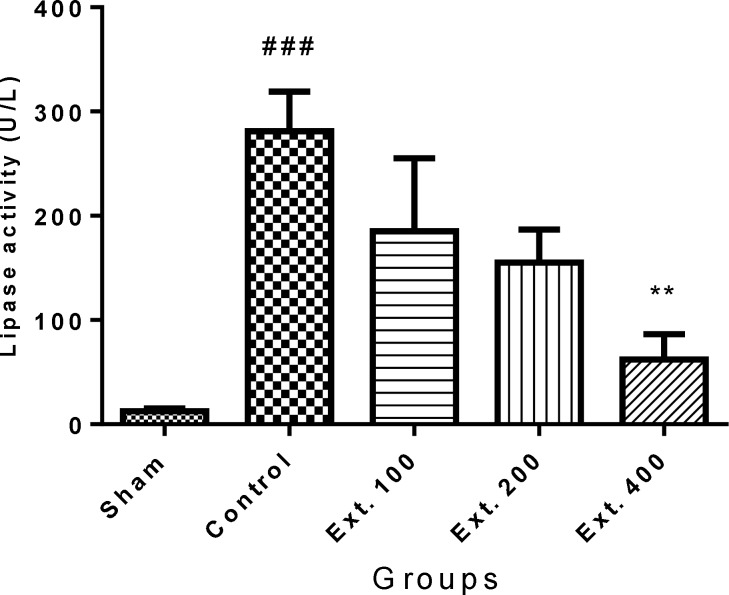
Effect of T. terrestris fruits hydro-alcoholic extract on serum lipase activity (U/L) of cerulein-induced acute pancreatitis in mice (i.p.). Sham: normal mice treated with normal saline (10 mL/kg); Control: received cerulein (50 µg/kg) and treated with normal saline (10 mL/kg), Ext. 100, 200, 400: received cerulein (50 µg/kg) and treated with extract, i.p.. Data are shown as mean±SEM of six animals in each group. ^**^p<0.01 versus control, ^###^ p<0.001 versus sham

Administration of cerulein (50 µg/kg, i.p.) caused a surge in amylase and lipase levels in the control group in comparison to sham group (p<0.001). As it is shown in Figures 1 and 2, administration of hydro-alcoholic extract of *T. terrestris *400 mg/kg (i.p.) caused a significant decline in serum level of amylase and lipase (p<0.01). 

**Figure 3:**
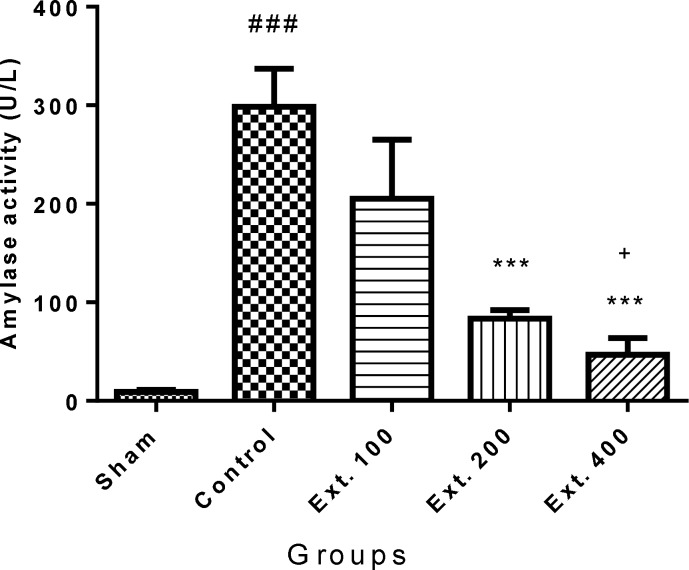
Effect of T. terrestris fruits hydro-alcoholic extract on serum amylase activity (U/L) of cerulein-induced acute pancreatitis in mice (p.o.) Sham: normal mice treated with normal saline (10 mL/kg); Control: received cerulein (50 µg/kg) and treated with normal saline (10 mL/kg), Ext. 100, 200, 400: received cerulein (50 µg/kg) and treated with extract, p.o.; Data are shown as mean±SEM of six animals in each group. ^*^^**^p<0.001 versus control, ^###^ p<0.01 versus sham, + p<0.05 versus Ext.100

**Figure 4:**
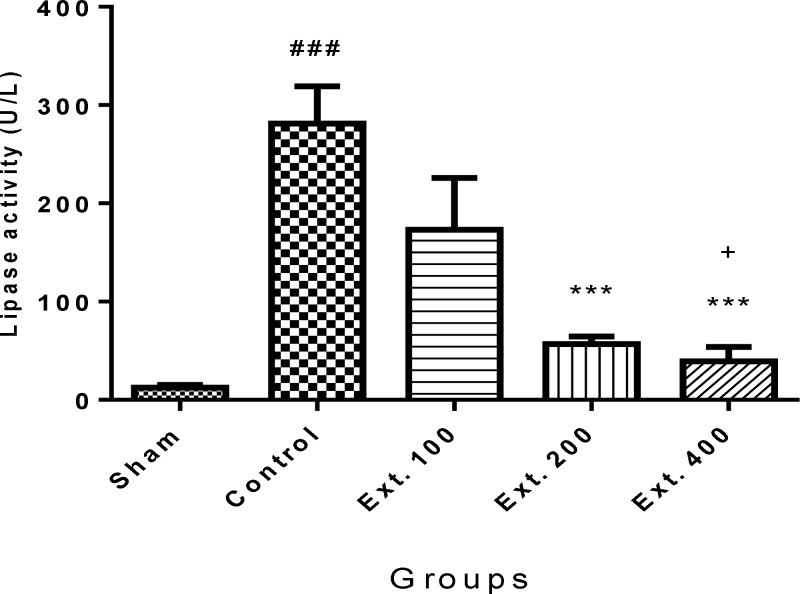
Effect of T. terrestris fruits hydro-alcoholic extract on serum lipase activity (U/L) of cerulien-induced acute pancreatitis in mice (p.o.) Sham: normal mice treated with normal saline (10 mL/kg); Control: received cerulein (50 µg/kg) and treated with normal saline (10 mL/kg), Ext. 100, 200, 400: received cerulein (50 µg/kg) and treated with extract, p.o.; Data are shown as mean±SEM of six animals in each group. ^***^p<0.001 versus control, ^###^ p<0.01 versus sham, + p<0.05 versus Ext.100

Besides, oral administration of *T. terrestris *extract 200 and 400 mg/kg markedly decreased both amylase and lipase activity (p< 0.001) (Figures 3 and 4).

**Effects of** ***T.*** ***terrestris* extract on the MPO activity**

**Figure 5:**
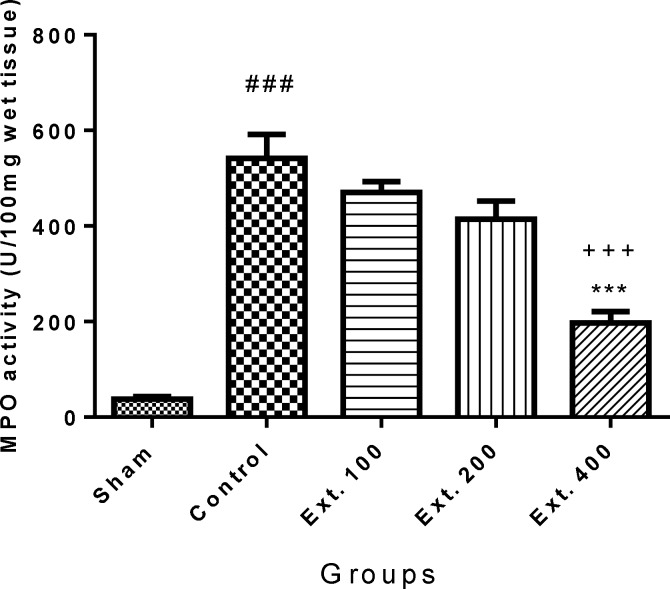
Effect of T. terrestris fruits hydro-alcoholic extract on myeloperoxidase activity (U/g) of cerulien-induced acute pancreatitis in mice (i.p.) Sham: normal mice treated with normal saline (10 mL/kg); Control: received cerulein (50 µg/kg) treated with normal saline (10 mL/kg); Ext. 100: treated with extract at dose of 100mg/kg i.p.; Ext. 200: treated with extract at dose of 200mg/kg i.p.; Ext. 300: treated with extract at dose of 300 mg/kg i.p.; Data are shown as mean±SEM of six animals in each group. ^***^p<0.001 versus control, ^###^ p<0.01 versus sham, ^+++^ p<0.001 versus Ext.100

**Figure 6:**
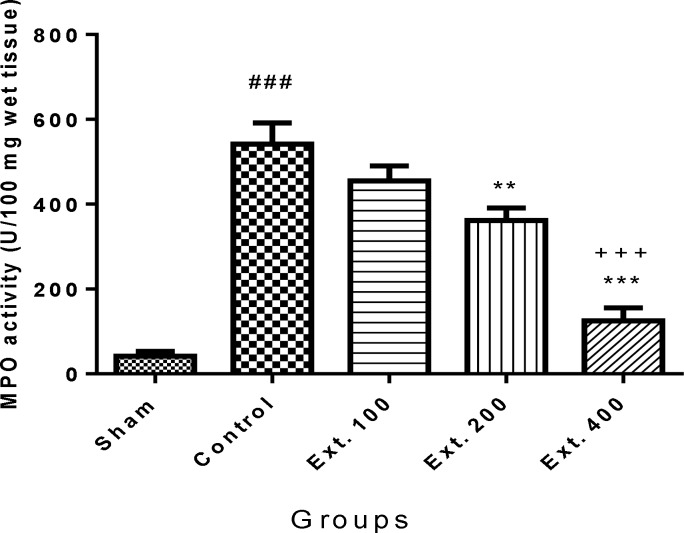
Effect of T. terrestris fruits hydro-alcoholic extract on myeloperoxidase activity (U/g) of cerulien-induced acute pancreatitis in mice (p.o.) Sham: normal mice treated with normal saline (10 mL/kg); Control: received cerulein (50 µg/kg) treated with normal saline (10 mL/kg); Ext. 100: treated with extract at dose of 100mg/kg i.p.; Ext. 200: treated with extract at dose of 200mg/kg i.p.; Ext. 300: treated with extract at dose of 300 mg/kg i.p.; Data are shown as mean ± SEM of six animals in each group. ^**^p<0.01, ^***^p<0.001 versus control, ^###^ p<0.001 versus sham, ^+++^ p<0.001 versus Ext.100

As it is shown in Figures 5 and 6, pancreatic MPO activity was increased in control group (p<0.001) following induction of pancreatitis. Pre-treatment with *T. terrestris* extract 400 mg/kg produced a significant reduction in MPO levels in both i.p. and p.o. treated groups (p<0.001). Besides, treatment of mice with *T. terrestris *extract 200 mg/kg, attenuated MPO activity level just in orally treated mice (Figure 6).

**Effects** ***T.*** ***terrestris* on the histological parameters**

**Figure 7:**
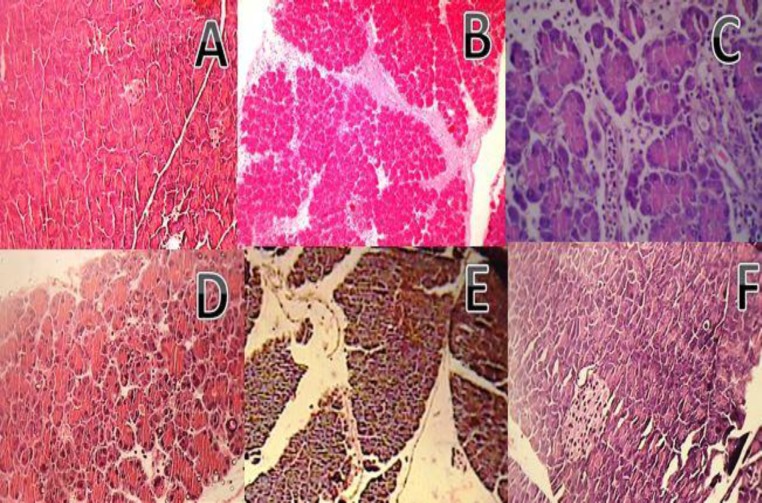
Microscopic illustration of pancreatic tissue in mice. Representative hematoxylin/eosin (H and E) sections of pancreas; (A×40) Normal tissue (intact pancreatic cells and acinar ducts); (B×10) and (C×40) Pancreatitis induced by cerulein (Inflammation, vacuolization, leukocyte infiltration and edema are visible). (D×40) Pancreatitis tissue treated with extract of T. terrestris at the dose of 200 mg/kg i.p. (E×40) (F×40) Pancreatitis tissue treated with extract of T. terrestris at the doses of 400 mg/kg i.p. and 400 mg/kg p.o., respectively showed significantly less histological alterations

 As shown in Figure 7a, sham group exhibited a normal architecture with intact epithelium in pancreatic tissue. On the other hand, the pancreas was grossly swollen and enlarged with a visible collection of edematous fluid in pancreatitis control tissue (Figures 7b and c). Upon microscopic examination, edema, hemorrhage, focal acinar necrosis, conspicuous vacuo1ization, and PMN infiltration in the pancreas were observed in the cerulean-treated control group (Figure 7b and c). In groups treated with *T.terrestris *extracts (200 and 400mg/kg) improvement in histopathology markers are detectable at different degrees (Figure 7d, e and f).

As shown in table 1, treatment with *T.terrestris *extract (200 mg/kg, i.p.) attenuated leukocyte infiltration in injurious pancreatic tissue compared to control group (p<0.05). Edema and leukocyte infiltration, two inflammatory indices were also decreased following administration of *T. terrestris *extract 400 mg/kg (p<0.05 and p<0.01, respectively) in pancreatic injurious mice. 

In groups treated with *T. terrestris *extract by oral route, administration of extract 200 mg/kg decreased leukocyte infiltration index in inflamed pancreatic tissue compared to control (p<0.05). Also, treatment of animals with 400 mg/kg of the extract significantly decreased edema (p<0.05) and leucocyte infiltration (p<0.01) index in treated groups (Table 2).

There was no significant change in vacuolization among the groups (Tables 1 and 2).

**Table 1:** Effect of T. terrestris fruits hydro-alcoholic extract on pathological scores of pancreas in cerulein-induced acute pancreatitis in mice (i.p.)

**Sham**	**0.0(0-0)**	**0.0(0-0)**	**0.0(0-0)**
**Control**	3.0## (1-3)	2.5## (1-3)	2.0## (1-3)
**Ext 100**	3.0(1-3)	2.5(1-3)	1.0 (0-3)
**Ext 200**	1.0(0-3)	1.0*(0-2)	2.0(1-3)
**Ext 400**	0.5*(0-2)	0.0** (0-1)	0.5(0-2)

Sham: normal mice treated with normal saline (10 mL/kg); Control: received cerulein (50 µg/kg) treated with normal saline (10 mL/kg); Ext. 100: treated with extract at dose of 100 mg/kg i.p.; Ext. 200: treated with extract at dose of 200 mg/kg i.p.; Ext. 300: treated with extract at dose of 300 mg/kg i.p.; Data are shown as median (range) of six animals in each group.

* p<0.05,

** p<0.01 versus control,

## p<0.01 versus sham,

++ p<0.01 versus Ext 100.

**Table 2:** Effect of T. terrestris fruits hydro-alcoholic extract on pathological scores of pancreas in cerulein-induced acute pancreatitis in mice (p.o.)

**Sham**	**0.0(0-0)**	**0.0(0-0)**	**0.0(0-0)**
**Control**	3.0## (1-3)	3.0## (1-3)	2.0# (0-3)
**Ext 100**	1.5(1-3)	2.0(0-3)	2.0(0-3)
**Ext 200**	1.0*(0-2)	0.5(0-1)	1.5(1-2)
**Ext 400**	0.5** ^+^ (0-1)	0.0**(0-2)	1.0(0-2)

Sham: normal mice treated with normal saline (10 mL/kg); Control: received cerulein (50 µg/kg) treated with normal saline (10 mL/kg); Ext. 100: treated with extract at dose of 100 mg/kg p.o.; Ext. 200: treated with extract at dose of 200 mg/kg p.o.; Ext. 300: treated with extract at dose of 300 mg/kg p.o.; Data are shown as median (range) of six animals in each group.

* p<0.05,

** p<0.01 versus control,

# p<0.05,

## p<0.01 versus sham,

+ p<0.05 versus Ext.100.

## Discussion

 The results of the present study clearly demonstrated that administration of *T. terrestris* hydro-alcoholic extract at doses of 200 and 400 mg/kg (p.o.) and at the dose of 400 mg/kg (i.p.) attenuated the severity of cerulein-induced pancreatitis in mice. This beneficial effect was indicated by biochemical, immunological, and histological evaluations.

 Our findings showed that *T. terrestris* decreased MPO activity, and serum amylase and lipase levels, and improved histological parameters. Moreover, this effect was likely dependent on the dose and route of administration. It is assumed that the dose of hydro-alcoholic extract is important for its effectiveness. It means higher doses possess more effective protection activity against AP while the lower doses have negligible or insignificant effect. 

The other influential parameter is the route of administration as it is manifested by the results, orally-treated mice had better response to treatment and it could be a consequence of locally active herb's components which are probably non-absorbable through the gastrointestinal (GI) tract.

Cerulein, is an analog of cholecystokinin (CCK) which acts as an agonist for CCK1 and CCK2 receptors, so, it activates Jak/Stat pathway and generates free oxygen radicals by inducing oxidant sensitive transcription factor (Hamilton et al., 1984 ▶). In cerulein-induced pancreatitis, reactive oxygen species are produced, resulting in accumulation of destructive oxygen and conducting leukocyte and prostaglandin synthesis; So, it is reasonable that any active compound which is able to scavenge free radicals and suppress NFκB generation, diminishes the inflammatory response, and eventually improve AP (Kim, 2008 ▶). It has been shown that aqueous extract of *T. terrestris* can induce cell growth arrest and apoptosis by down-regulating NF-κB signaling (Kim et al., 2011 ▶); So, inhibiting NF-κB signaling pathways in the pancreatic tissue could be one of the mechanisms by which this extract alleviated inflammation in AP.

The major chemical ingredients of * T. terrestris* are biologically active phenolic compounds like furostanol and spirostanol saponins which can trap free radicals and exhibit anti-oxidant activity (Zheleva-Dimitrova et al., 2012 ▶).

 Several studies have recognized a relationship between antioxidant activity and the amount of polyphenolic compounds within the herbs (Katalinic et al., 2006 ▶; Shan et al., 2005 ▶; Zheng and Wang, 2001 ▶). In this study, total phenolic content determination of extract showed that the amount of total phenols within the *T. terrestris *was significantly high that can confirm this correlation. It is probable that these compounds can protect pancreatic tissue against free oxydoradicals produced by cerulein. 

Antibacterial effect of *T. terrestris* has been reported by Kostova and Dinchev (2005) ▶. We know that AP could be associated with systemic acute bacterial infections (Beger et al., 1997 ▶), so this property of *T. terrestris* could support its application in a clinical setting. Anti-infection effect of *T. terrestris* has been attributed to DNA gyrase inhibiting property of its flavonoid contents and/or detergent-like saponins found in high amount within the extract (Bedir et al., 2002 ▶; Cushnie and Lamb, 2005 ▶; Lakhanpal and Rai, 2007 ▶). Khalid and his coworkers demonstrated that many phyto-constituents with anti-inflammatory effect were present in *T. terrestris* and the existence of 25 compounds was disclosed. A wide range of phytochemicals was responsible for anti-inflammatory activity of phenolic components (*i.e.* quercetin) and terpenoids (*i.e.* mono terpene lactone (−)-loliolide) that are tracked in this herb. Several mechanisms of action such as: (a) Antioxidative and radical scavenging activities (Ojha et al., 2006 ▶); (b) modulation of cellular activities of inflammation-related cells (mast cells, macrophages, lymphocytes, and neutrophils)(Mishra et al., 2013 ▶); (c) modulation of pro-inflammatory enzyme activities such as phospholipase A2 (PLA2), cyclooxygenase (COX), and lipoxygenase (LOX) and the nitric oxide (NO) producing enzyme, nitric oxide synthase (NOS) (Hong et al., 2002 ▶), have been proposed to explain the anti-inflammatory actions of these phyto-constituents.

 Because AP is one of the most prominent inflammatory diseases of the GI tract (Beger et al., 1997 ▶), it could be suggested that these mentioned mechanisms can modulate the severity of the disease. 

On the other hand, *T. terrestris* extract has a high capacity to suppress experimental colitis as indicated by the macroscopic, microscopic and biochemical evaluations carried out by Rajesh et al., (2013) ▶. The authors elucidated anti-IBD (inflammatory bowel disease) properties of *T. terrestris* in an animal model of colitis induced by dextran sulfate sodium (DSS). It seems that the inhibition of neutrophil infiltration into the colonic mucosa by *T. terrestris* suppressed the inflammatory responses, which leads to the development of DSS-induced colitis (Rajesh et al., 2013 ▶); since the pathologic mechanisms underlying AP and IBD are basically similar (Rasmussen et al., 1999 ▶), anti-colitis property of *T. terrestris* could explain its beneficial effect in AP.

In addition, it has been shown that *T. terrestris* has some more beneficial effects on serum lipid profile which is regularly deteriorated in AP. It reduced TG and LDL levels in experimental diet-induced hyperlipidemia (Chu et al., 2003 ▶).

Another beneficial action that has been elucidated for *T. terrestris*, is its analgesic effect. This effect that has been reported by Heidari et al. (2007) ▶ could alleviate the pain that is regularly associated with AP while this natural compound lacks ulcerogenic property of common NSAIDS and glucocorticoids (Graham, 2000 ▶; Heidari et al., 2007 ▶) in GI tract. 

In conclusion, the hydro-alcoholic extract of *T. terrestris* used in this study was potent enough to protect against an experimental model of pancreatitis induced by cerulein in mice. This property, could be attributed to many active ingredients that are present in this miraculous fruit. 

Several bioactivity and biological mechanisms of actions have been investigated and demonstrated for them; however, more mechanistic experiments are needed to identify the exact mechanisms that are involved.
